# Supplementary material for: GAGA zinc finger transcription factor searches chromatin by 1D–3D facilitated diffusion
Source: Nat Struct Mol Biol. 2025 Aug 5;32(11):2359–70. doi: 10.1038/s41594-025-01643-0 (PMC12618267; doi:10.1038/s41594-025-01643-0)
Supplement: Supplementary file 2 — Reporting Summary [file 41594_2025_1643_MOESM2_ESM.pdf]

nature portfolio

Corresponding author(s): Carl Wu, Taekjip Ha

Last updated by author(s): Jun 12, 2025

Reporting Summary

Nature Portfolio wishes to improve the reproducibility of the work that we publish. This form provides structure for consistency and transparency in reporting. For further information on Nature Portfolio policies, see our Editorial Policies and the Editorial Policy Checklist.

Statistics

For all statistical analyses, confirm that the following items are present in the figure legend, table legend, main text, or Methods section.

n/a	Confirmed	
☐	☒	The exact sample size (*n*) for each experimental group/condition, given as a discrete number and unit of measurement
☐	☒	A statement on whether measurements were taken from distinct samples or whether the same sample was measured repeatedly
☐	☒	The statistical test(s) used AND whether they are one- or two-sided<br>*Only common tests should be described solely by name; describe more complex techniques in the Methods section.*
☒	☐	A description of all covariates tested
☒	☐	A description of any assumptions or corrections, such as tests of normality and adjustment for multiple comparisons
☐	☒	A full description of the statistical parameters including central tendency (e.g. means) or other basic estimates (e.g. regression coefficient) AND variation (e.g. standard deviation) or associated estimates of uncertainty (e.g. confidence intervals)
☐	☒	For null hypothesis testing, the test statistic (e.g. *F*, *t*, *r*) with confidence intervals, effect sizes, degrees of freedom and *P* value noted<br>*Give P values as exact values whenever suitable.*
☒	☐	For Bayesian analysis, information on the choice of priors and Markov chain Monte Carlo settings
☒	☐	For hierarchical and complex designs, identification of the appropriate level for tests and full reporting of outcomes
☒	☐	Estimates of effect sizes (e.g. Cohen's *d*, Pearson's *r*), indicating how they were calculated

Our web collection on statistics for biologists contains articles on many of the points above.

Software and code

Policy information about availability of computer code

Data collection	smFRET data were collected using smCamera2 (https://github.com/Ha-SingleMoleculeLab/smCamera2). Optical tweezers and confocal microscopy data were collected using BlueLake software (LUMICKS).
Data analysis	smFRET data were analyzed using custom-written IDL and MATLAB scripts (https://github.com/ashleefeng/gaf_target_search; https://github.com/Yibenfu/HMM_3colorFRET); Optical tweezers and confocal microscopy data were analyzed using Pylake v.1.0.0 (https://lumicks-pylake.readthedocs.io/en/v1.0.0/theory/diffusion/diffusion.html). Graphs were plotted using OriginLab (https://www.originlab.com).

For manuscripts utilizing custom algorithms or software that are central to the research but not yet described in published literature, software must be made available to editors and reviewers. We strongly encourage code deposition in a community repository (e.g. GitHub). See the Nature Portfolio guidelines for submitting code & software for further information.

Data

Policy information about availability of data

All manuscripts must include a data availability statement. This statement should provide the following information, where applicable:

- Accession codes, unique identifiers, or web links for publicly available datasets
- A description of any restrictions on data availability
- For clinical datasets or third party data, please ensure that the statement adheres to our policy

All data are available within this manuscript and its supplementary information files. Raw data in "PMA" format for smFRET and "H5" format for 2D confocal scans and kymographs are available from the corresponding authors upon request due to the large file sizes.

Research involving human participants, their data, or biological material

Policy information about studies with human participants or human data. See also policy information about sex, gender (identity/presentation), and sexual orientation and race, ethnicity and racism.

Reporting on sex and gender	N/A
Reporting on race, ethnicity, or other socially relevant groupings	N/A
Population characteristics	N/A
Recruitment	N/A
Ethics oversight	N/A

Note that full information on the approval of the study protocol must also be provided in the manuscript.

Field-specific reporting

Please select the one below that is the best fit for your research. If you are not sure, read the appropriate sections before making your selection.

☒ Life sciences ☐ Behavioural & social sciences ☐ Ecological, evolutionary & environmental sciences

For a reference copy of the document with all sections, see nature.com/documents/nr-reporting-summary-flat.pdf

Life sciences study design

All studies must disclose on these points even when the disclosure is negative.

Sample size	For single-molecule experiments, 100-300 trajectories were obtained for each condition, typically from at least 3 individual experiments. No statistical analysis was used to predetermine sample sizes, which were chosen based on our past experience with these experiments (Panja et al. PMID 28825710, Hua et al. PMID 32913225).
Data exclusions	For single-molecule FRET experiments, traces were excluded if two or more peaks were evident. The exclusion criteria were pre-established in the field to address common technical issues such as optically overlapping molecules.
Replication	All experiments were performed at least 3 times in independent experiments. Replication was successful for all experiments.
Randomization	Not relevant to this study.
Blinding	Blinding was not relevant during data collection because our study did not involve human participants. Sample groups were not blinded during data analysis.

Reporting for specific materials, systems and methods

We require information from authors about some types of materials, experimental systems and methods used in many studies. Here, indicate whether each material, system or method listed is relevant to your study. If you are not sure if a list item applies to your research, read the appropriate section before selecting a response.

Materials & experimental systems

n/a	Involved in the study
☒	☐ Antibodies
☐	☒ Eukaryotic cell lines
☒	☐ Palaeontology and archaeology
☒	☐ Animals and other organisms
☒	☐ Clinical data
☒	☐ Dual use research of concern
☒	☐ Plants

Methods

n/a	Involved in the study
☒	☐ ChIP-seq
☒	☐ Flow cytometry
☒	☐ MRI-based neuroimaging

Eukaryotic cell lines

Policy information about cell lines and Sex and Gender in Research

Cell line source(s)	SF9 cell line was from the Eukaryotic Tissue Culture Facility at Johns Hopkins School of Medicine.
Authentication	SF9 cell line was not authenticated for this study.
Mycoplasma contamination	SF9 cells were not tested for mycoplasma contamination for this study.
Commonly misidentified lines (See ICLAC register)	N/A

Plants

Seed stocks	N/A
Novel plant genotypes	N/A
Authentication	N/A
