# Supplementary figures and images for: GAGA zinc finger transcription factor searches chromatin by 1D–3D facilitated diffusion
Source: Nat Struct Mol Biol. 2025 Aug 5;32(11):2359–70. doi: 10.1038/s41594-025-01643-0 (PMC12618267; doi:10.1038/s41594-025-01643-0)

Figure 4f

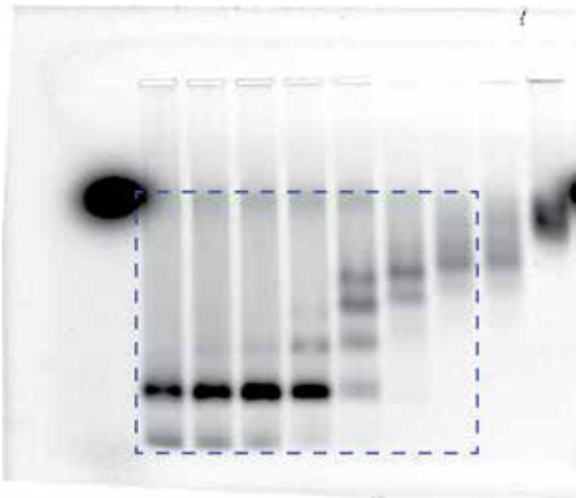

Figure 4g

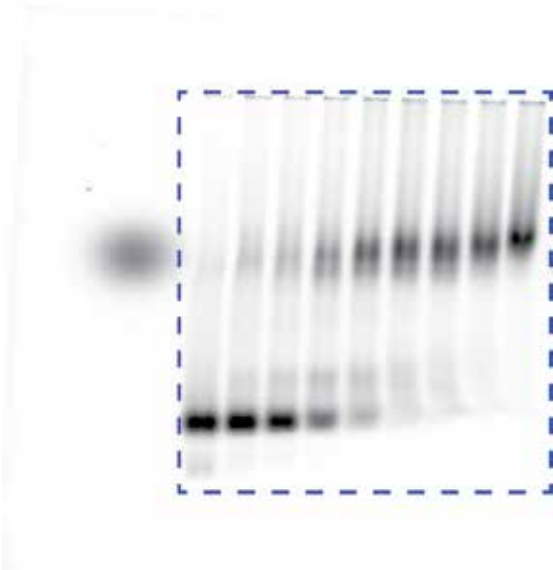

Figure 4h

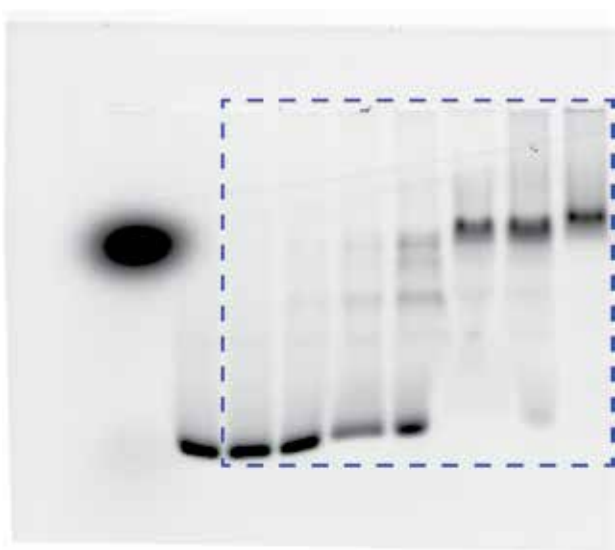

Supplement: Supplementary file 4 — Uncropped gels. [file 41594_2025_1643_MOESM4_ESM.pdf]

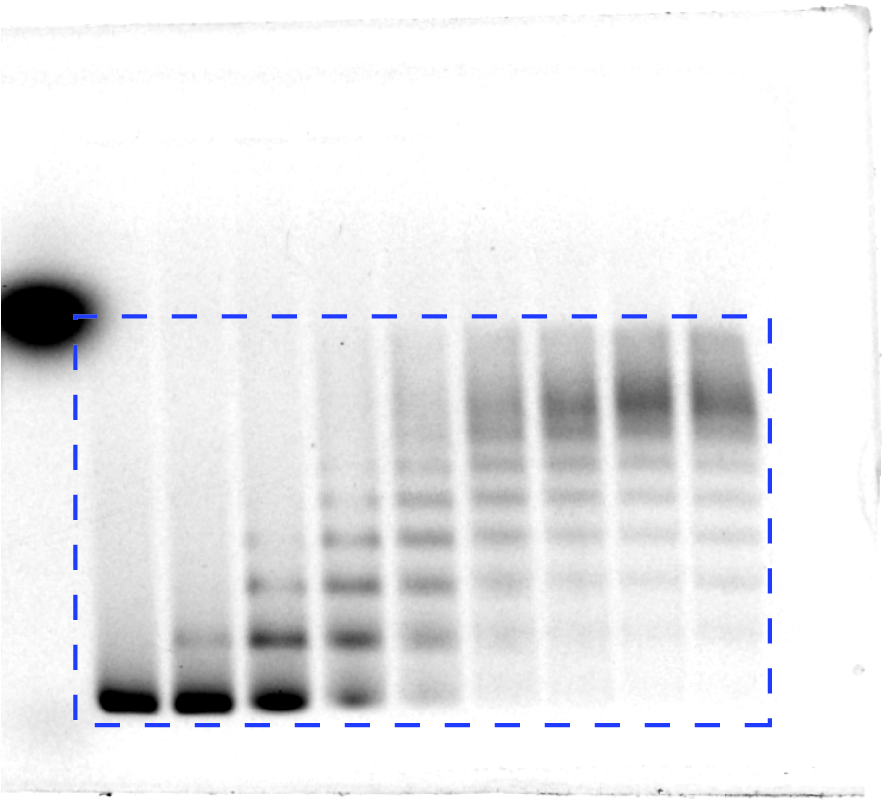

Supplement: Supplementary file 5 — Uncropped gels. [file 41594_2025_1643_MOESM5_ESM.tif]
