# Supplementary material for: GAGA zinc finger transcription factor searches chromatin by 1D–3D facilitated diffusion
Source: Nat Struct Mol Biol. 2025 Aug 5;32(11):2359–70. doi: 10.1038/s41594-025-01643-0 (PMC12618267; doi:10.1038/s41594-025-01643-0)

Extended Data Figure 6b

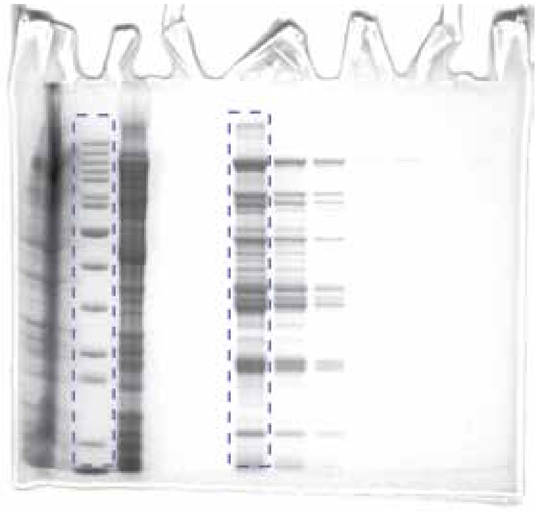

Extended Data Figure 6c

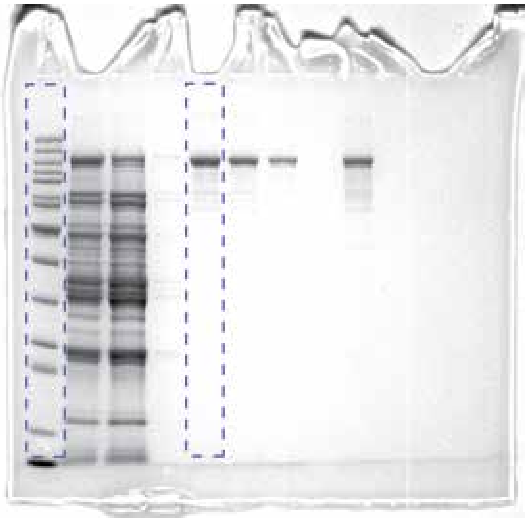

Extended Data Figure 6d

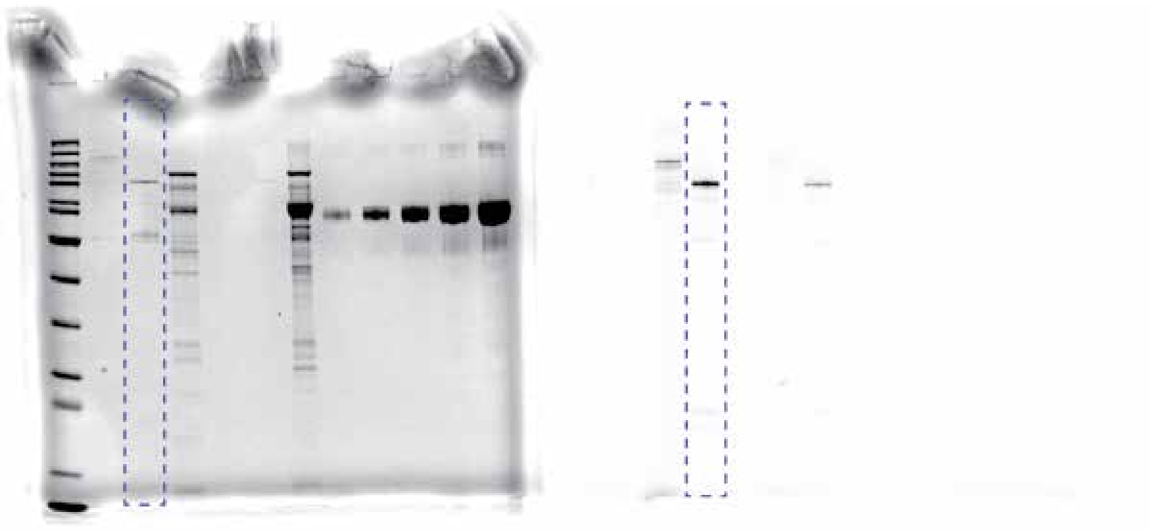

Coommassie

AlexaFluor 546 Fluorescence

Extended Data Figure 6e

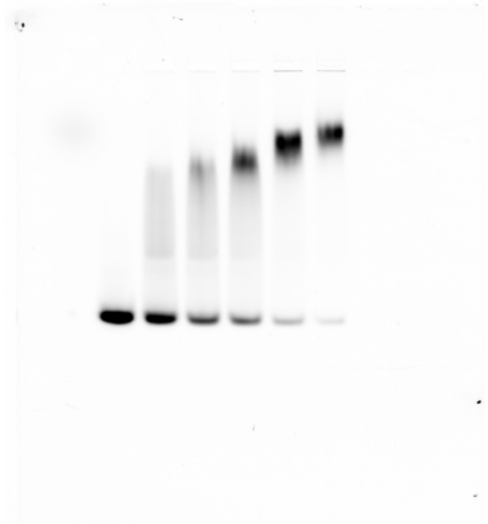

Supplement: Supplementary file 6 — Uncropped gels. [file 41594_2025_1643_MOESM6_ESM.pdf]
